# Pharmacokinetic and Pharmacodynamic Comparison of Chinese Herbal Ointment Liu-He-Dan and Micron Liu-He-Dan Ointment in Rats with Acute Pancreatitis

**DOI:** 10.1155/2014/389576

**Published:** 2014-02-16

**Authors:** Shi-Feng Zhu, Wei-Wei Chen, Jin Xiang, Xian-Lin Zhao, Mei-Hua Wan, Qin Yu, Mao-Zhi Liang, Wen-Fu Tang

**Affiliations:** ^1^Department of Integrative Medicine, West China Hospital, Sichuan University, Chengdu, Sichuan 610041, China; ^2^Department of Clinical Pharmacology, West China Hospital, Sichuan University, Chengdu, Sichuan 610041, China

## Abstract

*Aim*. To compare the pharmacokinetics and pharmacodynamics of herbal ointment Liu-He-Dan (LHD) and micron LHD (MLHD) in rats with acute pancreatitis (AP). *Methods*. Twenty rats were allocated into normal, AP, LHD, and MLHD groups. LHD or MLHD was applied on rats' abdomens. Plasma levels of emodin, rhein, aloe emodin, physcion, and chrysophanol were determined by high performance liquid chromatography—mass spectrometry—mass spectrometry (HPLC-MS-MS) at different time points, and the pharmacokinetic parameters were calculated. Serum amylase, TNF-**α**, IL-6, and IL-10 levels, and the pancreatic pathological scores were determined at 48 h after LHD or MLHD treatment. *Results*. *T*_1/2_ *α* and area under the curve (AUC) of emodin in the MLHD group were lower than those in the LHD group, while *T*_1/2_ *α* and AUC of aloe emodin in the MLHD group were higher than those in the LHD group (*P* < 0.05). *T*_1/2_ *α* and *T*_max_ of physcion in the MLHD group were significantly shorter than those in the LHD group (*P* < 0.05). Compared with the AP group, the amylase, malondialdehyde (MDA), TNF-**α**, and IL-6 levels decreased significantly after three days of treatment in LHD and MLHD groups, while the levels of superoxide dismutase (SOD), TNF-**α**, and the pancreatic pathological score, were similar. The pharmacodynamic parameters between the LHD and MLHD groups were similar. *Conclusion*. MLHD had better pharmacokinetics than, and similar pharmacodynamics to, LHD in the management of rats with AP, which indicated that MLHD might be substituted for LHD in the treatment of AP and thus reduce the amount of medicinal herbs used.

## 1. Introduction

Acute pancreatitis (AP) is a common and potentially lethal abdominal disease with high mortality [1]. Following the onset of AP, systemic inflammatory response syndrome (SIRS), as well as multiple organ dysfunction syndrome (MODS), might occur with ≥20% higher morbidity and mortality [2–6]. Over the last two decades, the treatment of AP has undergone fundamental changes based on new conceptual insights and evidence from clinical studies. Traditional Chinese Medicine (TCM) has played a key role in the conservative treatment for AP in China for more than 30 years. Previous studies showed that Da-Cheng-Qi decoction, the widely used TCM decoction, had beneficial effects on AP through promotion of gastrointestinal motility, anti-inflammatory effects, and pancreatic acinar apoptosis [7, 8].

In addition to oral administration and coloclysis with TCM decoction, external application of herbal preparations was also a common therapy for AP in West China Hospital (Sichuan University, China). Liu-He-Dan (LHD), a traditional herbal ointment, has been used to treat AP in West China Hospital for decades [9]. LHD helps to relieve pain and distension, promotes the absorption of pancreatic ascites, and prevents the formation of pseudocysts in patients with AP [10]. LHD consists of *Rheum officinale Baill*. (Polygonaceae), *Phellodendron chinense Schneid*. (Rutaceae), *Angelica dahurica* (Fisch. ex Hoffm.) Benth., Hook. f. ex Franch., and Sav. cv. Hangbaizhi. (Umbelliferae, *Armeniaca mume Siebold*. (Rosaceae), *Mentha haplocalyx Briq*., Labiatae), honey, flour, and other components (crude herbal medicinal proportions: 3 g, 3 g, 1.8 g, 1.5 g, 1.5 g, 1.8 g, 5 g, and 5 g). However, the preparation of crude LHD with coarse powder often leads to wasting of too many herbal resources, and the thick wrapped gauze, in addition to being uncomfortable for the patient, may lead to skin allergy, including rush and erythema. For these reasons, the traditional LHD was made into a fine powder of <200 microns in diameter and named micron LHD (MLHD) [11]. Our pervious studies demonstrated that AP significantly affected the pharmacokinetics of the absorbed components of LHD [12].

Thus, the present study aimed to compare the pharmacokinetics and pharmacodynamics of LHD and MLHD herbal ointments in rats with AP and provide a pharmacological basis for the clinical use of MLHD in patients with AP.

## 2. Materials and Methods

### 2.1. Animals

Sprague-Dawley (SD) male rats (*n* = 20) aged 90 ± 5 d with body weight of 320 ± 25 g were purchased from the Laboratory Animal Center of the West China Hospital. The animals were maintained at 22 ± 2°C in air-conditioned animal quarters with free access to water and standard laboratory rodent chow (Chengdu, China). After 1 week of acclimation, rats were fasted for 24 h before induction of AP and were kept under food-free conditions throughout the experiment. The animal study was performed according to the Guide for the Care and Use of Laboratory Animals of the National Institutes of Health. The protocol was approved by the Ethics Committee for Animal Experiments of our hospital.

### 2.2. Chemical Reagent and HPLC-MS-MS Conditions

All chemical sources (L-arginine, amylase, IL-6 and IL-10 ELISA-kits) were purchased from Chengdu Ronghai Chemical Reagent Factory (Chengdu, China). The LC-MS-MS system, including a SIL-HTc autosampler and a LC-10ADvp pump, was provided by Shimadzu (Kyoto, Japan).

### 2.3. Induction of Acute Pancreatitis

Rats were allocated into four groups: normal group (*n* = 5), AP model group (*n* = 5), AP model with LHD group (*n* = 5), and AP model with MLHD group (*n* = 5). After rats were anesthetized with ethyl ether, L-arginine (15 mg/kg BW) was injected into the abdominal cavity twice within 2 h to induce AP [13–15]. In normal groups, the same dose of saline was injected. Blood and pancreatic tissue samples were collected after the external application of LHD or MLHD at the indicated times [16].

### 2.4. Preparation of Chinese Herbal Ointments LHD and MLHD

Crude LHD powder was obtained from the pharmacy at our hospital (Chengdu, China) and authenticated by Professor WM Wang (Department of Herbal pharmacy, West China Hospital, Sichuan University, China). The crude herbal powder and four stainless steel balls were put into a PM-planetary ball mill of vacuum. The ball powder rate was set at 15 : 1 with a rotation speed of 200 r/min and a running time of 30 min [17]. Next, the traditional LHD was made into a fine powder of <200 microns in diameter and named micron LHD (MLHD) (yield = 65%). The components of LHD and MLHD ointments were determined by HPLC, with a reverse-phase C 18 column, and a mobile phase made up of methanol and 0.2% phosphoric acid (85 : 15, v/v). The ultraviolet detection was 254 nm. The quantities of aloe emodin, emodin, chrysophanol, rhein, and physcion were 0.18 ± 0.03 mg/g, 0.17 ± 0.03 mg/g, 0.31 ± 0.05 mg/g, 0.35 ± 0.05 mg/g, and 0.27 ± 0.04 mg/g in LHD and 0.16 ± 0.02 mg/g, 0.18 ± 0.03 mg/g, 0.34 ± 0.05 mg/g, 0.37 ± 0.04 mg/g, and 0.31 ± 0.05 mg/g in MLHD, respectively. The quantities of the five components were similar between the two formulas (*n* = 3). Flour, honey, and water were added into the herbal powder and mixed into a paste. Following the L-arginine injection, the same doses of LHD and MLHD ointments were applied topically on the rats' abdomens (application range: the xiphoid to the pubic syphilis, between the anterior axillary lines) [18–21]. The specimens (no. 20120915) were kept in our laboratory.

### 2.5. Plasma, Serum, and Pancreas Tissue Samples Collection

Blood samples (300 *μ*L) for analyzing the level of the five components (emodin, rhein, aloe emodin, physcion, and chrysophanol) from LHD and MLHD were collected from the eyes at 10 min, 30 min, 1 h, 2 h, 4 h, 8 h, 12 h, 24 h, and 48 h after the topical application of LHD or MLHD. After centrifugation at 3,000 r/min for 15 min, the supernatants were placed into heparinized tubes and stored at −80°C until analysis. Serum samples for analysis of amylase, TNF-*α*, IL-6, and IL-10 levels were collected 48 h after the topical application of LHD or MLHD. Pancreatic samples were obtained at 48 h following LHD or MLHD application to determine pancreatic pathological scores.

### 2.6. Determination of the Five Components from LHD, MLHD, Serum Amylase, and Inflammatory Cytokine Levels

Our previous study established a quantitative method to determine ten major components from Chinese herbal decoction Da-Cheng-Qi-Tang simultaneously by high performance liquid chromatography-mass spectrometry (HPLC-MS), including aloe emodin, emodin, chrysophanol, rhein, and physcion in rats and dogs. LHD and MLHD were composed of Dahuang (Radix et Rhizoma Rhei), Huang Bai (Cortex Phellodendri), Bai Zhu (Rhizoma Atractylodis Macrocephalae), Baizhi (Radix Angelicae Dahurcae), Wumei (Fructus Mume), Bohe (Herba Menthae), Feng Mi (Mel), and other components. As a pilot study, the present study used the established method of LC-MS-MS to detect the absorbed components of aloe emodin, emodin, chrysophanol, rhein, and physcion of Dahuang in rats after topical application of LHD and MLHD [22]. The level of amylase in plasma was determined with the iodide process according to the manufacturer's instructions. Serum levels of TNF-*α*, IL-6, and IL-10 were measured with ELISA kits according to the manufacturer's instructions.

### 2.7. Pathological Evaluation of the Pancreas

Pathological changes in the pancreatic tissue samples were scored as previously described [23]. In brief, pancreatic tissue samples were promptly collected at 48 h after topical application of LHD or MLHD, fixed in 10% neutral formalin, and embedded in paraffin. The paraffin-embedded pancreatic tissue blocks were cut into 5 mm thick sections and stained with hematoxylin and eosin. Specimens were scored by two independent pathologists blinded to the experimental setup using a scoring system for the extent and severity of pancreatitis (0–4, normal to severe, resp.), including the degree of interstitial edema, hemorrhage, necrosis, and inflammatory infiltration of the pancreatic tissue in each high-power field. The average score of ten fields was the final pathological score of the pancreas.

### 2.8. Data Collection and Statistical Analysis

Analyst 1.4.2 software for HPLC-MS was used for data collection, peak integration, and calibration. Concentrations of quality control and unknown samples were measured by interpolation from the calibration curves. Drug and statistics software programmed by the Chinese Pharmacological Society was used to process the plasma, pancreatic tissue concentration data, and compartment model fitting, after which all the pharmacokinetic parameters were determined, as follows: peak concentration (*C*_max⁡_), time of maximum plasma concentration (*t*_max⁡_), AUC (0–*t*), half-life (*t*_1/2_), and mean residence time (MRT).

All values were expressed as mean ± SD. All data were processed with statistical software PEMS 3.1. A *P* value of <0.05 was considered statistically significant.

## 3. Results

In the present study, aloe emodin, emodin chrysophanol, rhein, and physcion from Dahuang were measured by HPLC-MS. Only three of the components were successfully detected in rat plasma samples at the indicated time points after external application of LHD or MLHD on the abdomen. Chrysophanol and rhein were not detected in serum. The pharmacokinetic parameters of the detected emodin, physcion, and ale emodin from LHD and MLHD were calculated. The pharmacodynamic parameters of LHD and MLHD in AP were also compared.

### 3.1. Pharmacokinetics of Emodin from MLHD or LHD in Rats with AP

The *T*_1/2_ *α* and MRT of emodin in the MLHD group were significantly shorter than those in the LHD group (*T*_1/2_ *α*, *P* < 0.05; MRT, *P* > 0.05). The *T*_max⁡_ of emodin in the MLHD group was also significantly shorter than that in the LHD group (*P* > 0.05). Area under the curve (AUC) of emodin in the MLHD group was significantly lower than that in the LHD group (*P* < 0.05), while *C*_max⁡_ of emodin in the MLHD group was significantly higher than that in the LHD group (*P* > 0.05) (Table 1).

### 3.2. Pharmacokinetics of Aloe Emodin from MLHD or LHD in Rats with AP

The *T*_1/2_ *α* and *T*_max⁡_ of aloe emodin in the MLHD group were significantly longer than those in the LHD group (*T*_1/2_ *α*, *P* < 0.05; *T*_max⁡_, *P* > 0.05). The AUC and *C*_max⁡_ of aloe emodin in the MLHD group were significantly higher than those in the LHD group (AUC, *P* < 0.05; *C*_max⁡_, *P* > 0.05), while there was no significant difference in the serum MRT level of aloe emodin between the two groups (*P* > 0.05) (Table 1).

### 3.3. Pharmacokinetics of Physcion from MLHD or LHD in Rats with AP

The *T*_1/2_ *α* and *T*_max⁡_ of aloe Physcion in the MLHD group were significantly shorter than those in the LHD group (*T*_1/2_ *α*, *P* < 0.05; *T*_max⁡_, *P* < 0.05), and the AUC and *C*_max⁡_ of Physcion in the MLHD group were significantly lower than in those the LHD group (AUC, *P* > 0.05; *C*_max⁡_, *P* > 0.05). The MRT of Physcion in the MLHD group was significantly shorter than that in the LHD group (*P* > 0.05) (Table 1).

### 3.4. Concentration-Time Curves of the Three Components from LHD and MLHD

**Figure 1:**
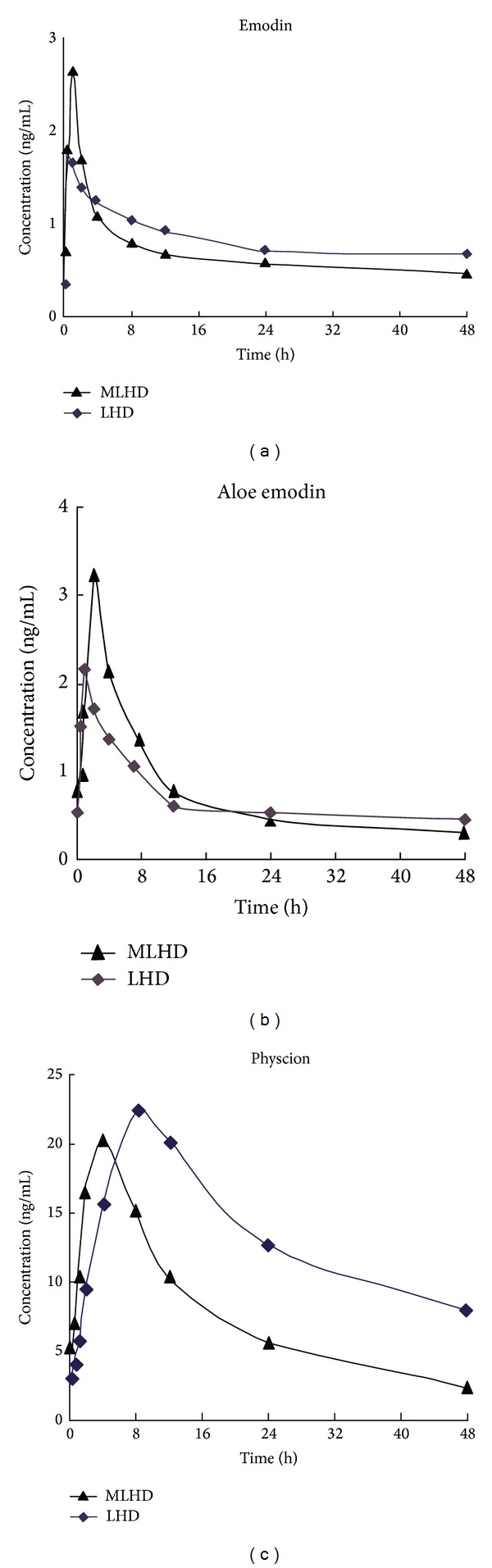
Estimated concentration-time curves of three components in rats with AP.

The concentration-time curves showed that the concentrations of the three components from MLHD in rats with AP were different from those in normal rats at different time points (see Figure 1).

### 3.5. Pharmacodynamics Comparison of LHD and MLHD in Rats with AP

As shown in Table 2, the levels of amylase, pancreatic malondialdehyde (MDA), and pathologic score in the AP group were higher than those in the normal group (*P* < 0.05), and the levels of pancreatic superoxide dismutase (SOD), serum TNF-*α*, and IL-6 in the AP model group were lower than those in the normal group (*P* < 0.05). After the LHD or MLHD treatment of AP rats, there was a significant decrease in amylase, MDA, TNF-*α*, and IL-6 levels (*P* < 0.05), while SOD was higher than that in the AP model group (*P* < 0.05). The levels of serum IL-10 were not significantly altered after the use of LHD and MLHD. These findings suggest that LHD and MLHD could help inhibit the inflammatory response of AP after external application on the abdomen. There was no statistical difference in the pathologic score among the LHD, MLHD, and AP model groups, which means that the effect of externally applied LHD or MLHD was not strong enough to alleviate the pancreatic pathological damage in rats with AP. There were no significant differences in all of the pharmacodynamic parameters between the MLHD and LHD groups. Overall, these results demonstrated that MLHD and LHD had a similar effect on the treatment of rats with AP.

**Figure 2:**
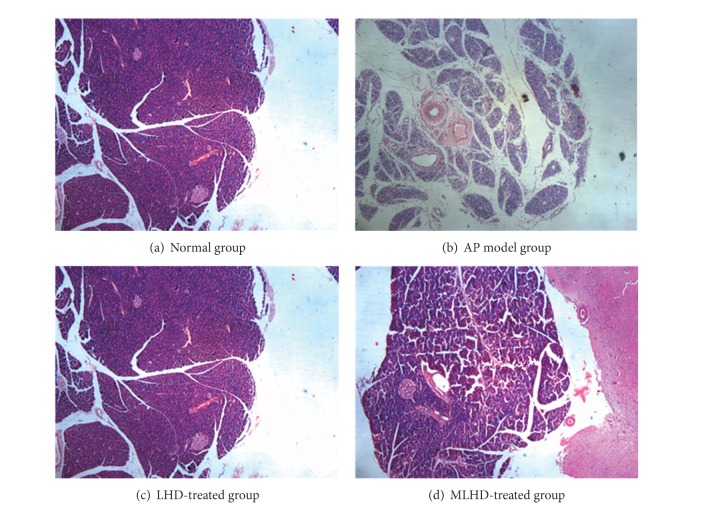
Pathological changes in the pancreas in different groups of rats stained with HE ×50.

As shown in Figure 2, the levels of pathologic injury in the AP group were more serious than those in the normal group, which means that AP model was constructed successfully. After the LHD or MLHD treatment of rats with AP, there was no obvious changes in the pancreatic pathology among the LHD, MLHD, and AP model groups, which means that the effect of externally applied LHD or MLHD within 72 h was not strong enough to alleviate the pancreatic pathological damage in rats with AP.

## 4. Discussion and Conclusions

The present study confirmed that there are significant differences in the pharmacokinetics of MLHD and LHD components and similar pharmacodynamics in rats with AP.

The *T*_1/2_ *α* of the MLHD components such as emodin and Physcion were significantly shorter than those of the LHD components (*T*_1/2_ *α*, *P* < 0.05), suggesting that efficient distribution, metabolism, and excretion of MLHD components can help reduce the drug accumulation *in vivo* and multiple dosing administration, leading to more stable drug concentrations and better therapeutic efficacy. In addition, the AUC of aloe emodin in MLHD was higher than that in LHD (*P* < 0.05), and the *T*_max⁡_ of Physcion in MLHD was shorter than that in LHD (*P* < 0.05). Even so, the other pharmacokinetic parameters of the three components were similar, including *C*_max⁡_ of emodin and aloe emodin in MLHD which were a little higher than those in LHD (*P* > 0.05). It was concluded that MLHD with higher AUC and higher *C*_max⁡_ may be superior to LHD in helping relieve the severity of AP.

Furthermore, the present study confirmed the similar effects of MLHD and LHD in the treatment of AP. Both LHD and MLHD could inhibit the inflammatory response by downregulating the expression of MDA, TNF-*α*, and IL-6 and upregulating SOD, resulting in the reduction of amylase. Unfortunately, this inhibitory effect was not strong enough to alleviate the pancreatic pathological damage. After treatment with LHD or MLHD, the levels of amylase, MDA, TNF-*α*, and IL-6 in both treatment groups were lower than those in the AP model group, and the level of SOD in both treatment groups was higher than that in the AP model group. We concluded that MLHD, similar to LHD, could also help ameliorate the severity of AP. Moreover, it could help decrease the level of proinflammatory cytokines such as IL-6 and TNF-*α* and suppress the inflammation in rats with AP, leading to the reduction of MDA, an increase of SOD, and, ultimately, the decrease in serum amylase. These results indicated that MLHD might be substituted for LHD in the treatment of AP.

Previous studies showed that the proinflammatory cytokine IL-6 and anti-inflammatory cytokine IL-10 are sensitive markers for the systematic inflammatory response in AP [24]. In this study, the levels of TNF-*α* and IL-6 in the LHD or MLHD treatment groups were lower than those in the AP model group, which suggested that MLHD (or LHD) could decrease IL-6 and TNF-*α* levels and suppress the inflammatory response in rats with AP. However, both LHD and MLHD did not upregulate the expression of IL-10, which contradicted some previous reports [25]. The possible reason for the discrepant results might be that the effect of MLHD or LHD was not strong enough to affect IL-10 and the pathologic score in the pancreas at 48 h after treatment or these herbal ointments could not regulate the inflammatory response through IL-10. A time- and dose-dependent study is needed to clarify this issue.

In summary, the three components of LHD and MLHD could be detected in rat serum after LHD and MLHD were externally applied on the abdomen. MLHD had better pharmacokinetics than, and similar pharmacodynamics to, LHD in the management of rats with AP, which indicated that MLHD might be substituted for LHD in the treatment of AP for the sake of saving herbal medicinal resources.

## Figures and Tables

**Table 1:** Pharmacokinetic parameters of LHD and MLHD components in AP model rats (*n* = 5).

Parameters	Emodin		Aloe emodin		Physcion	
	MLHD	LHD	MLHD	LHD	MLHD	LHD
*T*_1/2_ *α* (h)	1.14 ± 1*	5.8 ± 1.6	2.1 ± 0.4*	1.1 ± 0.6	1.7 ± 0.8*	3.9 ± 0.6
AUC (0–*t*) (ng/L∗h)	23.2 ± 8*	39.6 ± 9.2	44.6 ± 16.1*	21.2 ± 4.5	319 ± 76	437 ± 129
MRT (0–*t*) (h)	14.1 ± 1.4	16 ± 1.5	13.7 ± 0.6	13.6 ± 1.1	13.2 ± 1.1	14.5 ± 1.1
*T*_max⁡_ (h)	1.2 ± 0.45	0.8 ± 0.3	2.2 ± 1.1	1.5 ± 0.7	2.8 ± 1.1*	5.6 ± 2.2
*C*_max⁡_ (ng/L)	2.79 ± 1.39	2.24 ± 0.71	3.2 ± 0.9	2.08 ± 0.78	19.2 ± 9.5	24.2 ± 9.8

AUC: area under the curve; MRT: mean retention time; *compared to LHD group, *P* < 0.05.

**Table 2:** Pharmacodynamic parameters of LHD and MLHD in the pancreas at 48 h after topical use.

	Amylase (U/L)	MDA (nmol/mg)	SOD (U/mgprot)	TNF-*α* (pg/mL)	IL-6 (pg/mL)	IL-10 (pg/mL)	Pathologic score
Normal	1504 ± 372	0.03 ± 0.01	4.2 ± 1.5	0.06 ± 0.02	0.09 ± 0.03	0.37 ± 0.01	0.22 ± 0.22
AP model	4339 ± 611*	0.10 ± 0.03*	2.2 ± 1.1*	0.12 ± 0.05*	0.16 ± 0.04*	0.42 ± 0.04	5.63 ± 1.31*
LHD	2971 ± 730^#^	0.05 ± 0.02^#^	5.5 ± 1.9^#^	0.06 ± 0.01^#^	0.09 ± 0.03^#^	0.33 ± 0.06	5.53 ± 2.27
MLHD	2517 ± 244^#^	0.06 ± 0.02^#^	5.8 ± 2.1^#^	0.06 ± 0.01^#^	0.08 ± 0.02^#^	0.34 ± 0.03	5.75 ± 2.04

Compared with normal, *P < 0.05; compared with AP, ^#^ *P* < 0.05.

MDA: malondialdehyde; SOD: superoxide dismutase.

## Abbreviations

AP: Acute pancreatitis
LHD: Liu-He-Dan
MLHD: Micron Liu-He-Dan
HPLC-MS: High performance liquid chromatography-mass spectrometry
AUC: Area under the curve
MRT: Mean residence time
MDA: Malondialdehyde
SOD: Superoxide dismutase.
